# Supplementary material for: Centromere protection requires strict mitotic inactivation of the Bloom syndrome helicase complex
Source: Nat Commun. 2025 Aug 22;16:7832. doi: 10.1038/s41467-025-62966-6 (PMC12373852; doi:10.1038/s41467-025-62966-6)
Supplement: Supplementary file 1 — Supplementary Information [file 41467_2025_62966_MOESM1_ESM.pdf]

SUPPLEMENTARY INFORMATION

Centromere protection requires strict mitotic inactivation of the Bloom syndrome helicase complex

María Fernández-Casañas[1,4], Eleftheria Karanika[1,6], Umit Aliyaskarova[1,6], Tomisin Olukoga[1], Alex D Herbert[1], Antony W Oliver[2], Matthew Day[3], Adrijana Crncec[1,5] and Kok-Lung Chan[1*]

1. Chromosome Dynamics and Stability Group, Genome Damage and Stability Centre, University of Sussex, Brighton, BN1 9RQ, United Kingdom

2. DNA Repair Enzymes Group, Genome Damage and Stability Centre, University of Sussex, Brighton, BN1 9RQ, United Kingdom

3. Centre for Molecular Cell Biology, School of Biological and Behavioral Sciences, Blizard Institute, Queen Mary University of London, London, E1 2AT, United Kingdom

4. Present address: Division of Cell and Molecular Biology, The Institute of Cancer Research, London, SW3 6JB, United Kingdom

5. Present address: Laboratory of Cancer Biology and Genetics, Centre for Cancer Research, National Cancer Institute, Bethesda, MD, USA

6. These authors contributed equally: Eleftheria Karanika, Umit Aliyaskarova

*Correspondence: koklung.chan@sussex.ac.uk (K.L.C.)

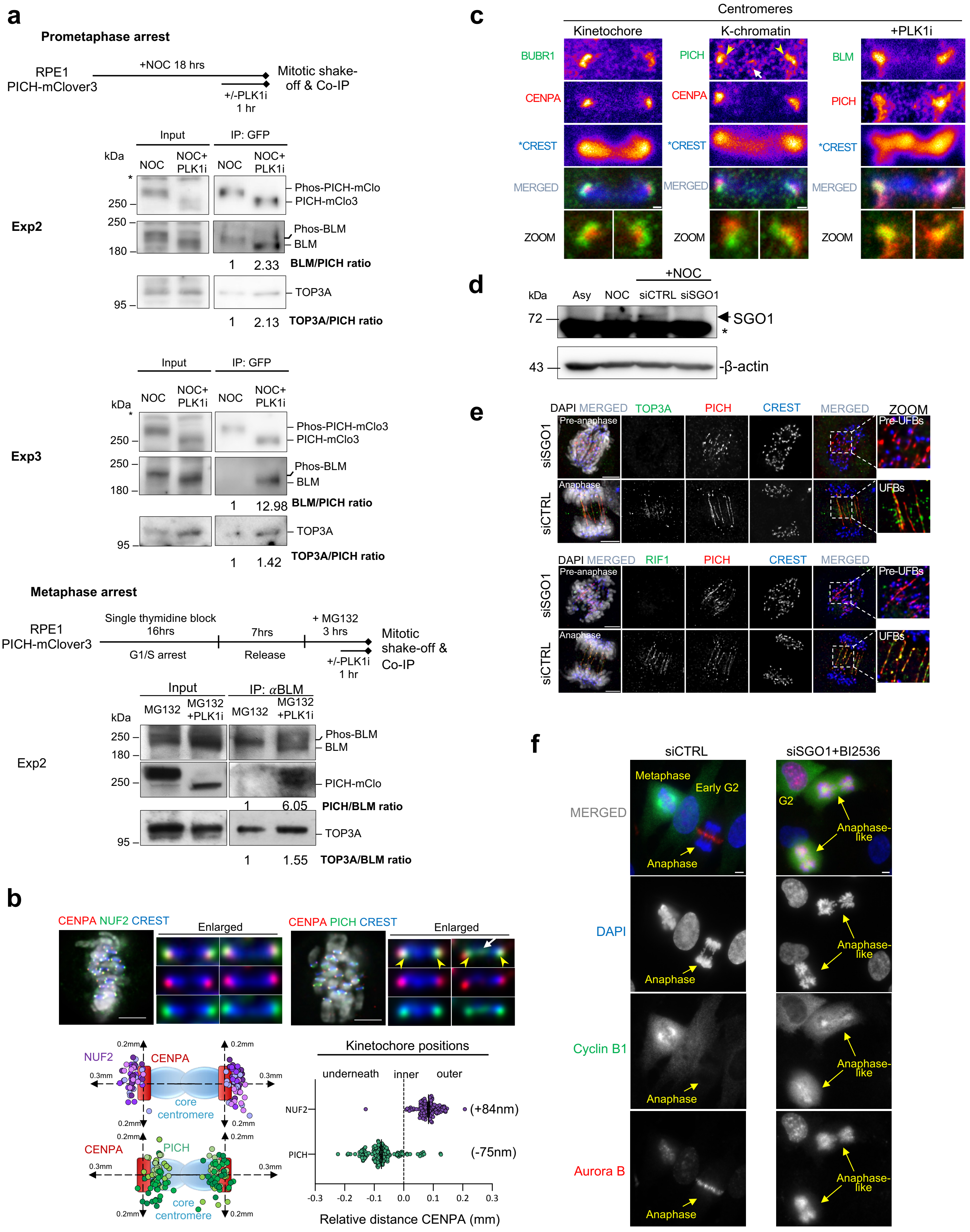
a
Prometaphase arrest
RPE1 PICH-mClover3
+NOC 18 hrs
+/-PLK1i 1 hr
Mitotic shake-off & Co-IP
Input
IP: GFP
kDa
NOC
NOC+PLK1i
Exp2
Phos-PICH-mClo
PICH-mClo3
Phos-BLM
BLM
1 2.33 BLM/PICH ratio
TOP3A
1 2.13 TOP3A/PICH ratio
Exp3
Phos-PICH-mClo3
PICH-mClo3
1 12.98 BLM/PICH ratio
1 1.42 TOP3A/PICH ratio
Metaphase arrest
Single thymidine block 16hrs
G1/S arrest
7hrs
Release
+ MG132 3 hrs
+/-PLK1i 1 hr
Mitotic shake-off & Co-IP
IP: αBLM
MG132
MG132 +PLK1i
PICH-mClo
1 6.05 PICH/BLM ratio
1 1.55 TOP3A/BLM ratio
b
CENPA NUF2 CREST
Enlarged
CENPA PICH CREST
NUF2
CENPA
PICH
core centromere
Kinetochore positions
underneath
inner
outer
(+84nm)
(-75nm)
Relative distance CENPA (mm)
c
Centromeres
Kinetochore
K-chromatin
+PLK1i
BUBR1
PICH
BLM
*CREST
MERGED
ZOOM
d
Asy
+NOC
siCTRL
siSGO1
SGO1
-β-actin
e
DAPI MERGED
TOP3A
RIF1
Pre-anaphase
Anaphase
Pre-UFBs
UFBs
siSGO1
siCTRL
f
siSGO1+BI2536
Metaphase
Early G2
G2
Anaphase-like
DAPI
Cyclin B1
Aurora B

Supplementary Figure 1. Limited binding of BLM and RIF1 with PICH on pre-UFB structures before anaphase.

a Repetition of co-immunoprecipitation of BLM, PICH-mClover3 and TOP3A in nocodazole- (top) or MG132-arrested (bottom) mitotic cells in the presence or absence of PLK1i treatments. Quantification of the normalised ratios are shown (See also Figure 1a).

b The precise localisation of PICH at human centromeres. Deconvolved images of RPE1 metaphase cells showing the localisation of NUF2 and PICH relative to CENPA. Scale bars, 5 µm. Measurements showing the relative distances of NUF2 and PICH to CENPA (numbers of foci measured, NUF2 n=114 and PICH n=150; mean ± S.E.M is shown). Arrowheads denote K-chromatin, and the arrow indicates UFB precursors.

c Super-resolution STED nanoscopy showing the localisation of PICH at K-chromatin (arrowheads) and UFB precursors (arrow), and the colocalisation of BLM and PICH after PLK1 inhibition. BUBR1, CENPA and PICH-mClover3 were imaged by STED whereas *CREST by confocal microscopy. Scale bars, 100 nm. A diagram illustrating the K-chromatin and UFB precursors bound by PICH but not the BTRR complex and RIF1. MT, microtubules; KT, Kinetochores; NUF2, Outer kinetochore marker; CENPA, inner kinetochore marker. Scale bars = 100nm

d Western blotting shows SGO1 depletion by RNA interference in RPE1 cells in asynchronous and nocodazole treated cells. The asterisk marks non-specific bands. b-actin acts as a loading control.

e Representative images showing TOP3A and RIF1 staining on pre-anaphase UFBs and anaphase UFBs in RPE1 cells pre-treated with the indicated siRNA oligos and ICRF193. PICH staining was used to label the UFBs structures and CREST for centromeres. Scale bars, 5 µm

f Representative images showing the staining of Cyclin B1 and Aurora B in siCTRL and PLK1i-treated SGO1-depleted RPE1 cells. Arrows indicate anaphase and anaphase-like cells, respectively. Scale bars, 5 µm.

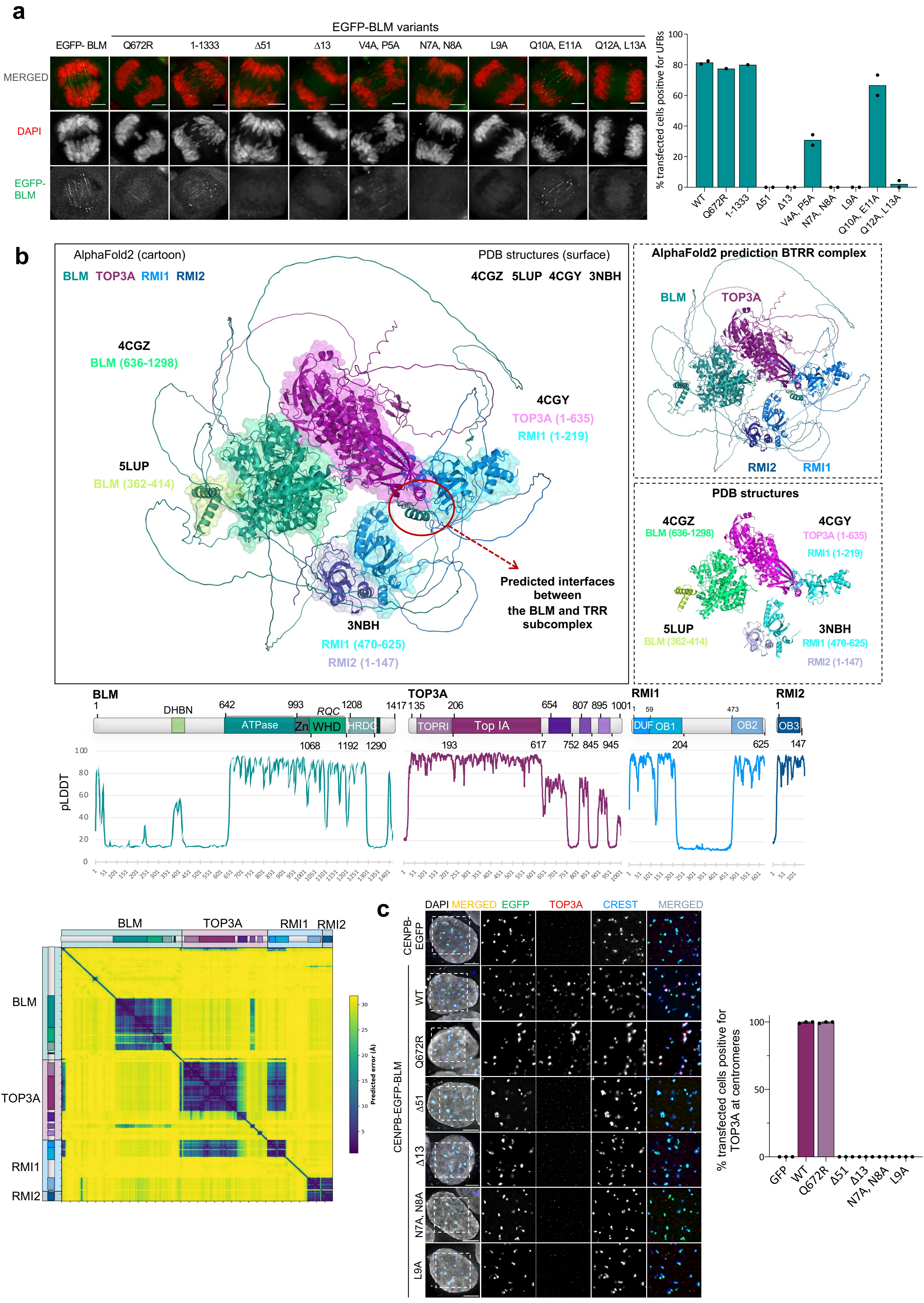
a
EGFP-BLM variants
EGFP- BLM
Q672R
1-1333
Δ51
Δ13
V4A, P5A
N7A, N8A
L9A
Q10A, E11A
Q12A, L13A
MERGED
DAPI
EGFP-BLM
% transfected cells positive for UFBs
WT
b
AlphaFold2 (cartoon)
BLM TOP3A RMI1 RMI2
PDB structures (surface)
4CGZ 5LUP 4CGY 3NBH
4CGZ
BLM (636-1298)
5LUP
BLM (362-414)
4CGY
TOP3A (1-635)
RMI1 (1-219)
3NBH
RMI1 (470-625)
RMI2 (1-147)
Predicted interfaces between the BLM and TRR subcomplex
AlphaFold2 prediction BTRR complex
BLM
TOP3A
RMI2
RMI1
PDB structures
BLM
DHBN
ATPase
Zn
WHD
RQC
HRDC
TOP3A
TOPRI
Top IA
RMI1
DUF
OB1
OB2
RMI2
OB3
pLDDT
Predicted error (Å)
c
DAPI MERGED EGFP TOP3A CREST MERGED
CENPB-EGFP
CENPB-EGFP-BLM
WT
Q672R
Δ51
Δ13
N7A, N8A
L9A
% transfected cells positive for TOP3A at centromeres
GFP

Supplementary Figure 2. BLM binds to TOP3A and RMI1 through two adjacent N-terminal motifs.

a HeLa cells were transiently transfected with EGFP-BLM variants, synchronised in G2/M by 9 μM RO-3306, and released in the presence of 1 μM ICRF193 to enrich anaphase populations containing UFBs. Representative images of BLM UFB localisation. Percentages of transfected anaphase cells having ≥ 1 EGFP-BLM UFB. Numbers of anaphase cells examined: WT n=40, 36; Δ51 n=45, 32; Δ13 n=44, 35; V4A,P5A n=40, 32; N7A,N8A n=42, 36; L9A n=46, 30; Q10A,E11A n=45, 30; Q12A,L13A n=33, 46 from two independent experiments, except in Q672R n=40 and 1-1333, n=45, from one experiment.

b Cartoon representations of the BTRR complex made by AlphaFold2 prediction and X-ray crystallography structures. PDB:4CGZ, BLM helicase domain; PDB:5LUP, double helical bundle in BLM N-terminus (DHBN); PDB: 4CGY, TOP3A (TOPRIM motif and the ATPase domain) and RMI1 (DUF and OB1-fold domain); PDB: 3NBH, RMI1 (OB2-fold domain) and RMI2 (OB3-fold domain). The red circle highlights the interfaces between BLM and the TRR subcomplex. Schematic representation of the subunits of the BTRR complex (DHBN: double helical bundle in N-terminus; RQC: RecQ C-terminal domain; WHD: Winged Helix domain; HRDC: Helicase and RNaseD C-terminal domain; DUF: domain of unknown function; OB: Oligonucleotide/Oligosaccharide-binding domain). pLDDT scores for each domain within the BTRR complex. The x-axis represents the residue position within each domain, while the y-axis represents the pLDDT score, indicating the predicted local distance difference between the model and experimental structure. The plot depicts the PAE (Predicted Aligned Error) values for residue-residue alignment within the subunits of the BTRR complex. PAE values are visualised using colour intensity.

c An in vivo BLM-TOP3A interaction assay. EGFP-BLM is tethered to centromeres in ΔBLM HAP1 cells by fusing with CENPB (1-158). Percentages of transfected cells positive for TOP3A staining at centromeres are shown. Numbers of transfected cells analysed from three independent experiments: EGFP n=50, 65, 61; EGFP-BLM WT n=86, 99, 102; EGFP-BLM Q672R n=72, 97, 85; EGFP-BLM Δ51 n=115, 88, 100; EGFP-BLM Δ13, n=97, 86, 106; EGFP-N7A, N8A n=86, 102, 104; EGFP-L9A n=98, 105, 75, mean ± S.D. is shown.

All Scale bars, 5 μm.

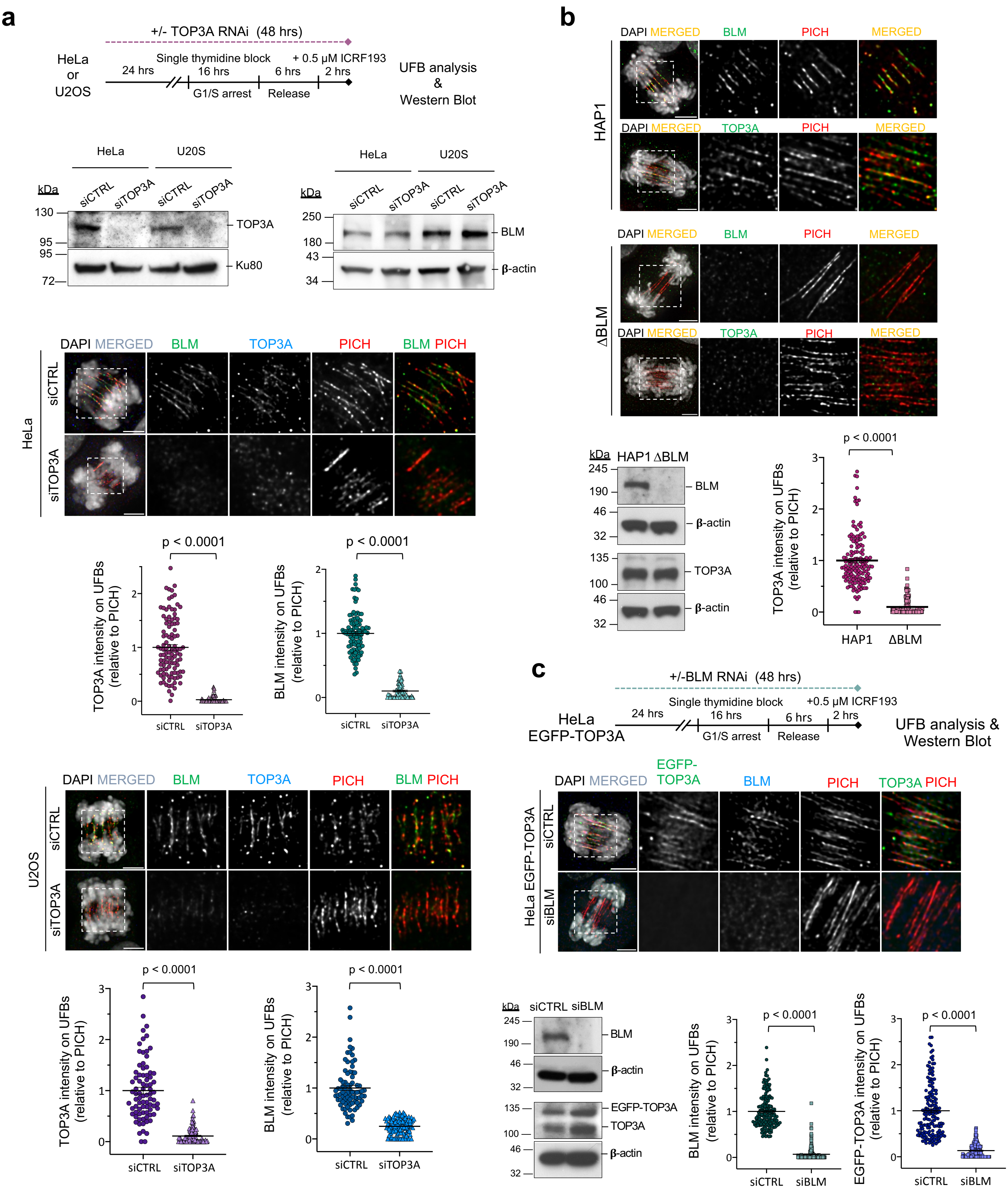
a
+/- TOP3A RNAi (48 hrs)
HeLa or U2OS
24 hrs
Single thymidine block 16 hrs
G1/S arrest
6 hrs
Release
+ 0.5 µM ICRF193 2 hrs
UFB analysis & Western Blot
HeLa
U20S
kDa
siCTRL
siTOP3A
TOP3A
Ku80
BLM
β-actin
DAPI MERGED
BLM
TOP3A
PICH
BLM PICH
siCTRL
siTOP3A
p < 0.0001
TOP3A intensity on UFBs (relative to PICH)
BLM intensity on UFBs (relative to PICH)
U2OS
b
DAPI MERGED
MERGED
HAP1
ΔBLM
HAP1 ΔBLM
TOP3A intensity on UFBs (relative to PICH)
c
+/-BLM RNAi (48 hrs)
HeLa EGFP-TOP3A
+0.5 µM ICRF193
UFB analysis & Western Blot
EGFP-TOP3A
TOP3A PICH
HeLa EGFP-TOP3A
siBLM
siCTRL siBLM
EGFP-TOP3A intensity on UFBs (relative to PICH)

Supplementary Figure 3. The binding of BLM and TOP3A proteins to anaphase UFBs is interdependent.

a Experimental outline of TOP3A RNA interference (RNAi) in HeLa and U2OS cells. TOP3A and BLM protein levels were analysed by western blotting after TOP3A knockdown. Representative immunofluorescence images of anaphase HeLa cells (upper panel) or U2OS cells (lower panel) showing BLM, TOP3A and PICH localisation on UFBs after siCTRL or siTOP3A oligos treatments. The graphs show the intensities of BLM or TOP3A relative to PICH on individual UFBs in both conditions. Data are normalised to the average intensity in the control condition. Numbers of UFBs measured from a single experiment in HeLa; siCTRL n=105, siTOP3A n=76 and U20S: siCTRL n=80, siTOP3A n=114; 12 anaphase cells were scored in each condition; mean ± S.E.M. is shown.

b Representative immunofluorescence images of wildtype and ΔBLM HAP1 anaphase cells stained for TOP3A and BLM on UFBs. PICH is used to label the UFBs. BLM and TOP3A protein levels were analysed by Western blotting in both cell lines. The relative TOP3A intensities (to PICH) on PICH-coated UFBs are shown. Data are normalised to the average intensity of TOP3A in wildtype HAP1 cells. Numbers of UFBs measured from a single experiment in HAP1 n=152 from 19 anaphases; in ΔBLM n=153 from 18 anaphases; mean ± S.E.M. is shown.

c Experimental outline of BLM RNAi in HeLa cells stably expressing EGFP-TOP3A. Representative immunofluorescence images of anaphase cells showing BLM, EGFP-TOP3A and PICH localisation to UFBs after siCTRL or siBLM oligos treatments. BLM and EGFP-TOP3A protein levels were analysed by Western Blot. The graphs show quantification of EGFP-TOP3A and BLM intensities relative to PICH. Data are normalised to the average intensity in the control condition. Numbers of UFBs measured in a single experiment: siCTRL n=163 from 17 anaphases; siBLM n=185 from 18 anaphases; mean ± S.E.M. is shown.

All scale bars, 5 μm. P values are calculated by unpaired two-tailed Welch's t-test.

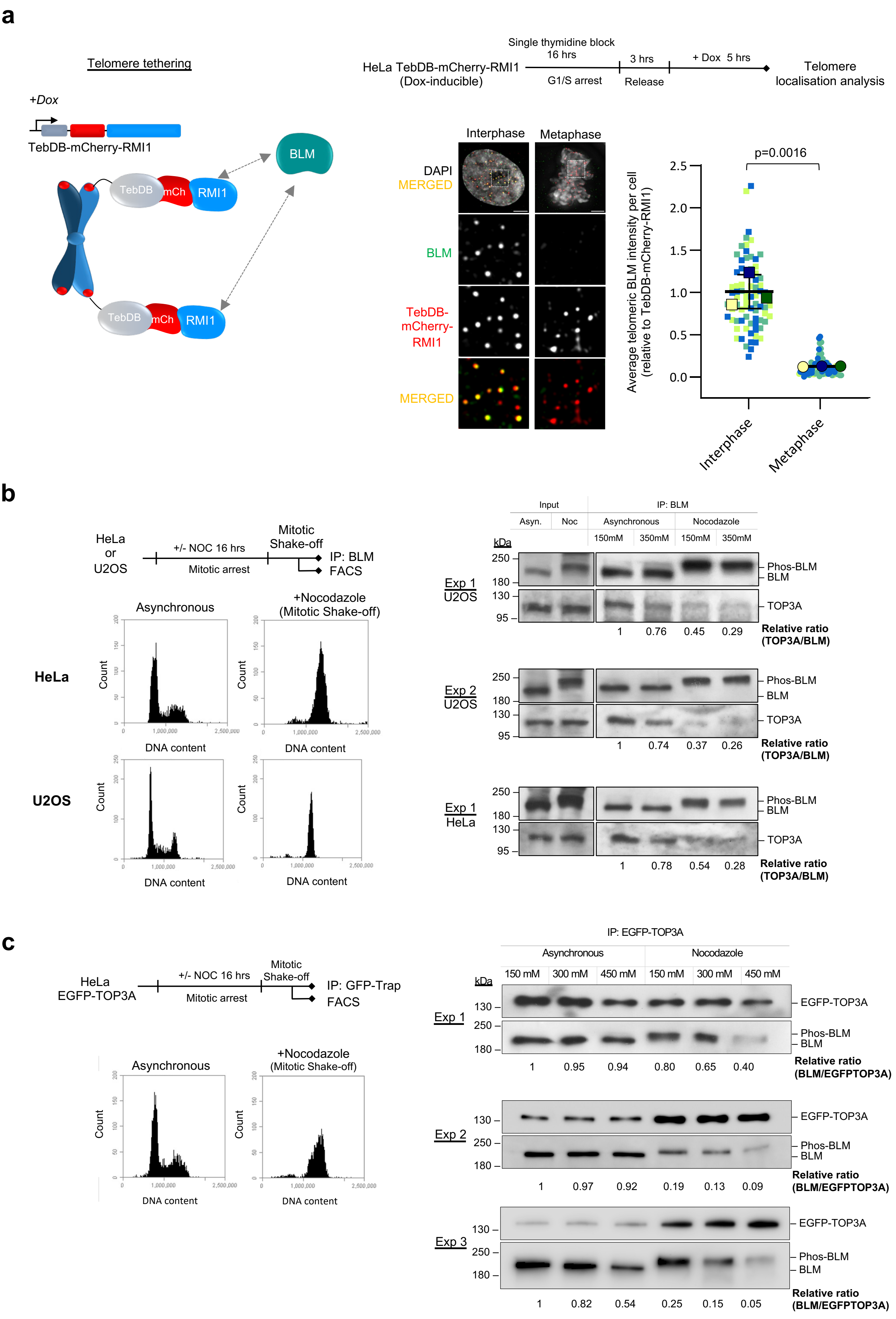

a
Telomere tethering
+Dox
TebDB-mCherry-RMI1
TebDB
mCh
RMI1
BLM
HeLa TebDB-mCherry-RMI1
(Dox-inducible)
Single thymidine block
16 hrs
G1/S arrest
3 hrs
Release
+ Dox 5 hrs
Telomere
localisation analysis
Interphase
Metaphase
DAPI
MERGED
BLM
TebDB-
mCherry-
RMI1
MERGED
Average telomeric BLM intensity per cell
(relative to TebDB-mCherry-RMI1)
p=0.0016
2.5
2.0
1.5
1.0
0.5
0.0
Interphase
Metaphase
b
HeLa
or
U2OS
+/- NOC 16 hrs
Mitotic arrest
Mitotic
Shake-off
IP: BLM
FACS
Asynchronous
+Nocodazole
(Mitotic Shake-off)
HeLa
U2OS
Count
DNA content
Input
IP: BLM
Asyn.
Noc
Asynchronous
Nocodazole
150mM
350mM
150mM
350mM
kDa
Exp 1
U2OS
250
180
130
95
Phos-BLM
BLM
TOP3A
1
0.76
0.45
0.29
Relative ratio
(TOP3A/BLM)
Exp 2
U2OS
1
0.74
0.37
0.26
Relative ratio
(TOP3A/BLM)
Exp 1
HeLa
1
0.78
0.54
0.28
Relative ratio
(TOP3A/BLM)
c
HeLa
EGFP-TOP3A
+/- NOC 16 hrs
Mitotic arrest
Mitotic
Shake-off
IP: GFP-Trap
FACS
Asynchronous
+Nocodazole
(Mitotic Shake-off)
Count
DNA content
IP: EGFP-TOP3A
Asynchronous
Nocodazole
150 mM
300 mM
450 mM
150 mM
300 mM
450 mM
kDa
Exp 1
130
250
180
EGFP-TOP3A
Phos-BLM
BLM
1
0.95
0.94
0.80
0.65
0.40
Relative ratio
(BLM/EGFPTOP3A)
Exp 2
1
0.97
0.92
0.19
0.13
0.09
Relative ratio
(BLM/EGFPTOP3A)
Exp 3
1
0.82
0.54
0.25
0.15
0.05
Relative ratio
(BLM/EGFPTOP3A)

Supplementary Figure 4. BLM-TOP3A interaction becomes unstable during mitosis.

a An in vivo system to study RMI1 and BLM interaction in HeLa cells. mCherry-tagged RMI1 was immobilised at telomeres via TebDB fusion (left). Telomeric intensities of BLM (relative to TebDB-mCherry-RMI1) were measured in interphase and metaphase cells (right). Numbers of cells measured from three independent experiments: Interphase n=26, 24, 27; metaphase n=30, 33, 32; mean + S.D. is shown. The p value is calculated by unpaired two-tailed t-test. Scale bars = 5mm.

b Asynchronous and mitotic shake-off cells were collected after the indicated treatments in HeLa and U2OS cells followed by immunoprecipitation of endogenous BLM. FACS (Fluorescent Activated Cell Sorting) profiles of different samples are shown (left). The immunoprecipitated protein extracts were subjected to washing buffer containing different salt (NaCl) concentrations, as indicated, and analysed by Western Blot (right). Quantification of the normalised TOP3A/BLM ratio is shown per experiment.

c Similar to **b** except that GFP trap co-immunoprecipitation was performed in HeLa cells stably expressing EGFP-TOP3A. Quantification of the normalised BLM/EGFP-TOP3A ratio is shown per experiment.

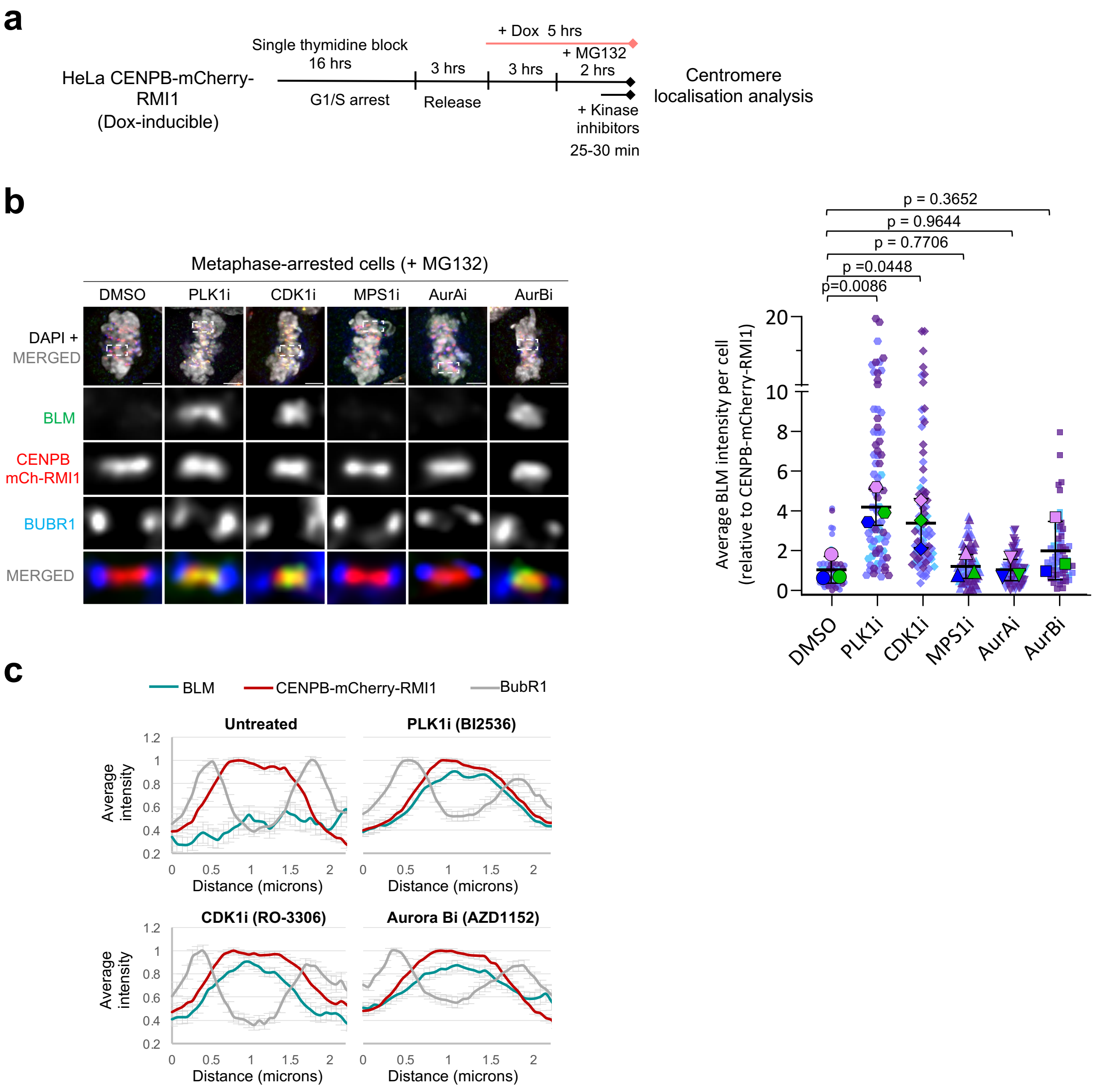

Supplementary Figure 5. Acute PLK1 or CDK1 inhibition restores BLM interaction with centromere-tethered RMI1 in metaphase cells.

a Experimental outline of centromere localisation analysis under different mitotic kinase inhibition in HeLa metaphase cells expressing CENPB-mCherry-RMI1.

b Representative images of HeLa metaphase-arrested cells stained for endogenous BLM, CENPB-mCherry-RMI1 and the outer kinetochore marker, BubR1. Scale bars, 5 µm. Enlarged images illustrate a single centromere. The graph shows quantification of average centromeric intensity of BLM relative to CENPB-mCherry-RMI1 per cell. Data is normalised to the average intensity in DMSO condition. Numbers of cells analysed from three independent experiments: DMSO n=13, 28, 24; PLK1i n=12, 38, 28; CDK1i n=13, 35, 30; MPS1i n=12, 38, 24; AurAi n=11, 37, 28; AurBi n=14, 25, 29. mean ± S.D. is shown.

c Scanline analyses showing BLM binding is enhanced within the core centromere regions Numbers of centromeres analysed per condition, DMSO n=22; PLK1i n=21; CDK1i n=23, AurBi n=24. All p values are calculated by unpaired two-tailed t-test.

a

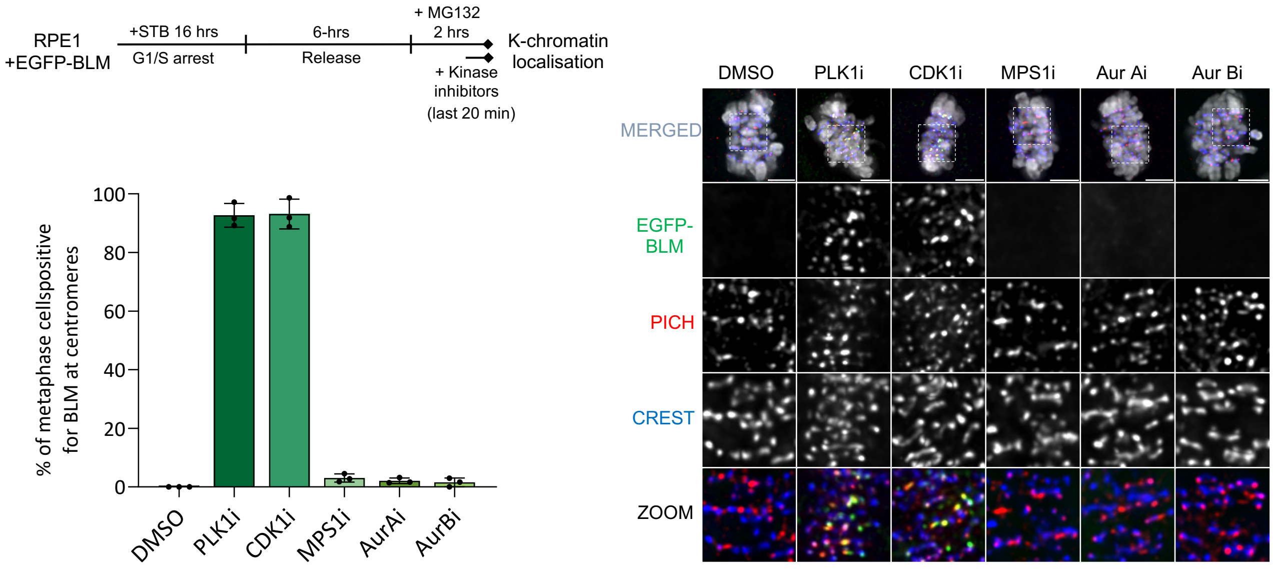

b

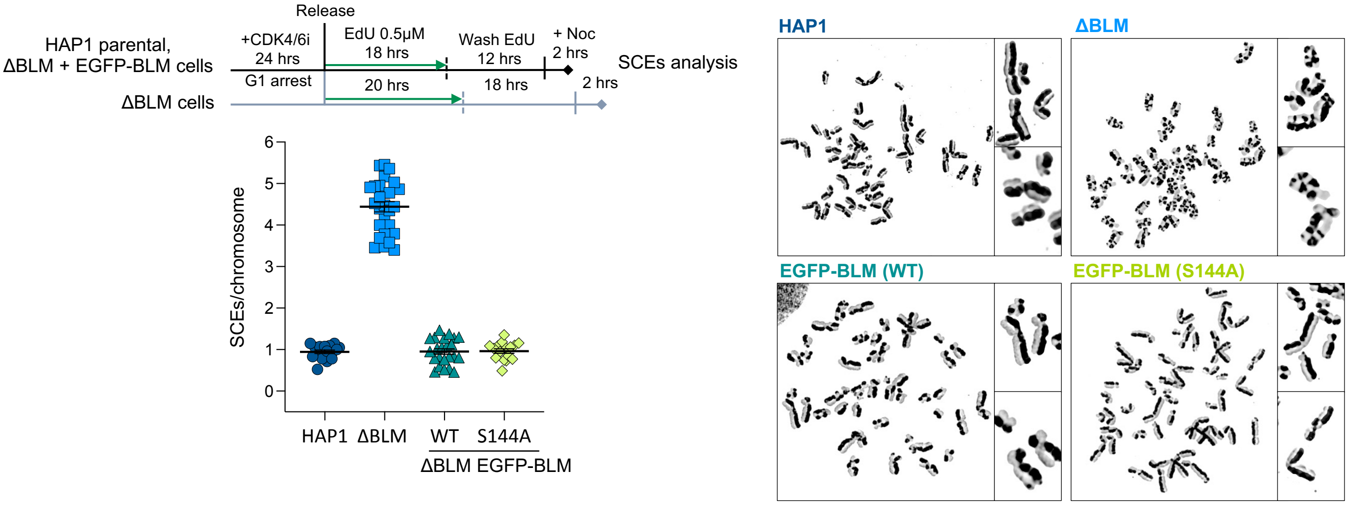

c

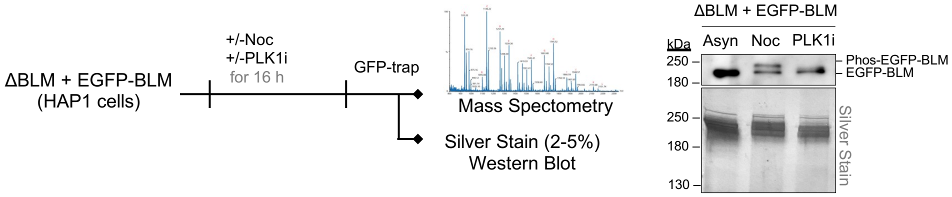

			PLK1i (M/S)	Mitotic-arrested cells				PLK1 inhibition/depletion		
**BLM**	Phosphopeptides	GPS-Polo	BI2536	Nocodazole	Noscapine	Nocodazole	Nocodazole	BI4834	BI2536	TAL / shRNA
S26	TLNNKL**S**LSKPKFSGFT									
S48	SDNNV**S**VTNVSVAKTPVL			Mitotic	Mitotic					
T50	SDNNVSV**T**NVSVAKTPVL									
S53	SDNNVSVTNV**S**VAKTPVL			Mitotic						
S72	RNKDVNVTEDF**S**FSEPLPNT			Mitotic						
S269	HLEDERDN**S**EKKKNLEE		Phosphorylated				Mitotic			
S282	NLEEAELH**S**TEKVPC		Dephosphorylated		Mitotic					
S336	DTSDRKEDVL**S**TSKDLL									
T337	DTSDRKEDVLS**T**SKDLL					Mitotic				
S338	DTSDRKEDVLST**S**KDLL		Dephosphorylated			Mitotic				
S358	MSMQELNPET**S**TDCDARQ	Consensus		Mitotic	Mitotic					
S367	STDCDARQI**S**LQQQLIHVM		Dephosphorylated							
S422	LTEVDFNKSDA**S**LLGSLWRY		Phosphorylated	Mitotic		Mitotic				
S464	SHLPSNSV**S**PGDCLL		Phosphorylated			Mitotic				
S539	KDQNKHTA**S**INDLER		Dephosphorylated		Mitotic	Mitotic				
			Current study	Dephoure, 2008. PNAS	Malik, 2009. JPR	Olsen, 2010. Sci Signal	Hegemann, 2011. Sci Signal	Grosstessner-Hain, 2011. MCP	Kettenbach, 2011. Sci Signal	Santamaria, 2011. MCP

Supplementary Figure 6. PLK1 and CDK1 prevent the localisation of BLM to K-chromatin in early mitosis.

a Experimental outline of cell synchronisation for K-chromatin localisation analysis in RPE1 cells stably expressing EGFP-BLM. Representative images showing metaphase-arrested cells after treatment with the indicated kinase inhibitors. PICH staining is used to mark the K-chromatin and CREST for centromeres. Quantification of the percentage of cells positive for BLM at K-chromatin. Numbers of metaphase cells analysed from three independent experiments: DMSO n=62, 64, 70; PLK1i n= 65, 70, 60; CDK1i n=86, 75, 80; MPS1i n=72, 66, 55; AurAi n=68, 62, 60; AurBi n=60, 67, 45; mean ± S.D. is shown. All scale bars, 5 μm.

b Experimental outline of cell synchronisation and EdU labelling for SCEs analysis in HAP1, ΔBLM and ΔBLM cells complemented with EGFP-BLM WT and S144A mutant. Graph shows quantification of SCE frequency per chromosome, per metaphase spread. Each data point represents the average number of SCEs per chromosome in one spread. (Numbers of metaphases analysed from a single experiment, HAP1 n=17; ΔBLM n=27; ΔBLM+WT BLM n=23; ΔBLM +S144A n=16; mean ± S.E.M is shown). Representative SCE images of metaphase spreads of each cell line.

c Identification of PLK1-dependent phosphorylation in mitotic BLM. Experimental setup for mass spectrometry of the immunoprecipitated BLM proteins from ΔBLM EGFP-BLM cells in the presence or absence of nocodazole/PLK1 inhibitor treatment. Western Blot and silver stain show the enriched EGFP-BLM proteins, and the electrophoretic mobility shifts in the indicated conditions. Systematic analysis of PLK1-mediated phosphorylation on BLM: Table shows BLM sites dephosphorylated (sensitive) or phosphorylated (induced) upon PLK1 inhibition (BI2536) identified in the current study. Phosphopeptides sequences are shown, as well as sites predicted to be phosphorylated by Polo-like kinases (PLKs) using bioinformatic software Group-based Prediction System (GPS-Polo). Summary of BLM sites identified in previous proteome-wide analyses (references are depicted) of PLK1-regulated phosphorylation sites in mitotic human cells; either mitotic-specific sites using Nocodazole or Noscapine, or PLK1-dependent phosphorylation sites using shRNA or small-molecule inhibitors of PLK1: BI4834, BI2536 or TAL (ZK-Thiazolidinone).

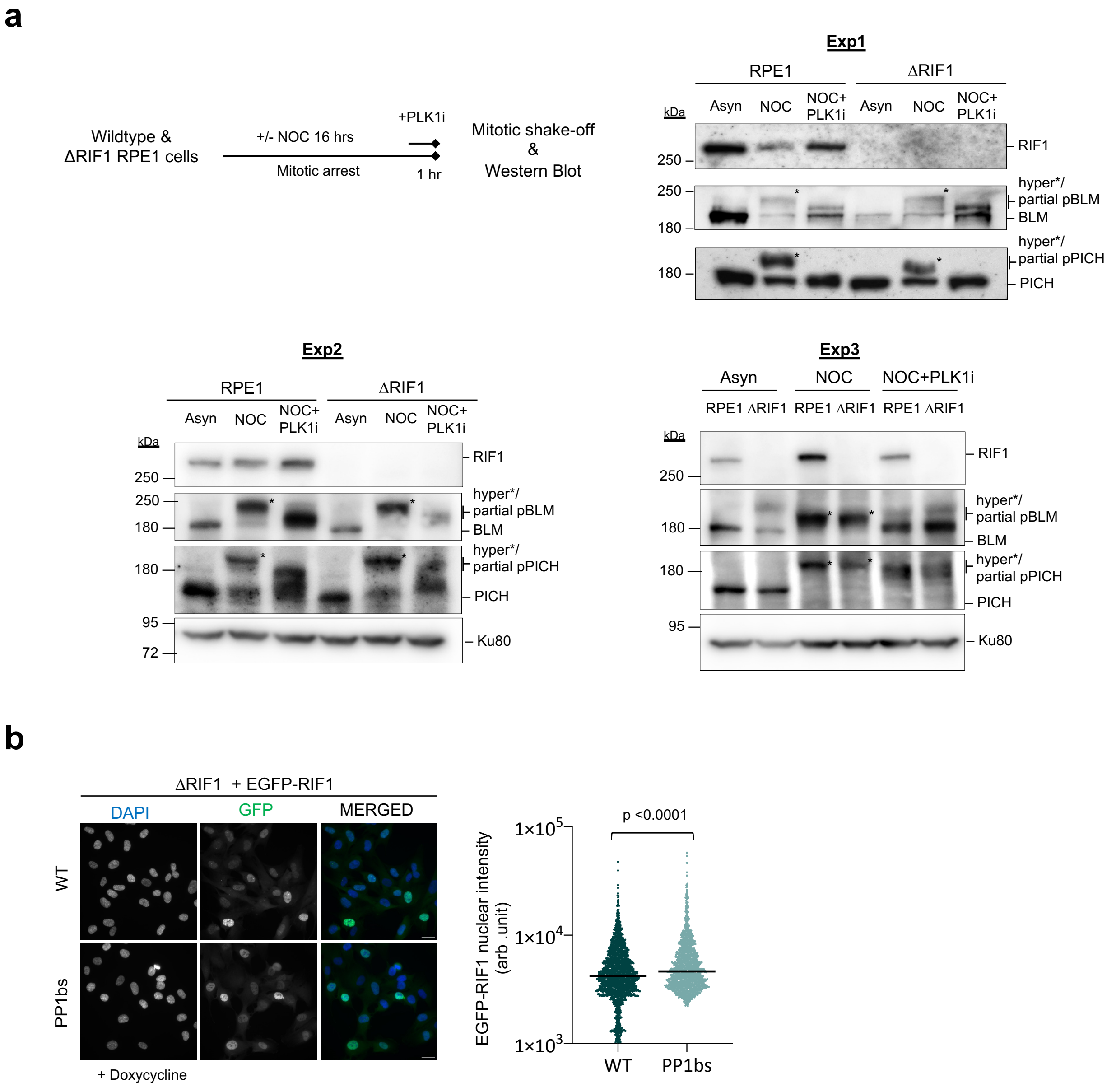

Supplementary Figure 7. RIF1 ablation does not prevent the loss of hyperphosphorylation of BLM and PICH after PLK1 inhibition.

a Wildtype and RIF1 knockout RPE1 cells were arrested in mitosis by 50ng/ml of nocodazole for 16 hrs. 100nM of BI2536 was added at the last 1 hour followed by mitotic shake-off and Western blotting analysis. Asterisks denote the hyperphosphorylation forms of BLM and PICH. Three independent experiments were performed.

b Characterisation of ΔRIF1 RPE1 cells with Dox-inducible wildtype (WT) or PP1s-binding mutant (PP1bs) of EGFP-RIF1. The levels of nuclear RIF1 were measured by quantitative immunofluorescence imaging (total numbers of nuclei scored from one experiment: WT n=1840; PP1bs n=1479).
