# Supplementary material for: Centromere protection requires strict mitotic inactivation of the Bloom syndrome helicase complex
Source: Nat Commun. 2025 Aug 22;16:7832. doi: 10.1038/s41467-025-62966-6 (PMC12373852; doi:10.1038/s41467-025-62966-6)
Supplement: Supplementary file 2 — Description of Additional Supplementary Files [file 41467_2025_62966_MOESM2_ESM.pdf]

Description of Additional Supplementary Files

File Name: Supplementary Movie 1

Description: Centromeric localisation of EGFP-TOP3A (green) in HeLa cells expressing CENPB-mCherry-RMI1 fusion protein (red).

File Name: Supplementary Movie 2

Description: Centromeric localisation of EGFP-TOP3A (green) in HeLa cells expressing CENPB-mCherry-BLM(Q672R) fusion proteins (red).
