# Supplementary material for: Centromere protection requires strict mitotic inactivation of the Bloom syndrome helicase complex
Source: Nat Commun. 2025 Aug 22;16:7832. doi: 10.1038/s41467-025-62966-6 (PMC12373852; doi:10.1038/s41467-025-62966-6)
Supplement: Supplementary file 5 — Reporting Summary [file 41467_2025_62966_MOESM5_ESM.pdf]

nature portfolio

Corresponding author(s): Kok-Lung Chan
Last updated by author(s): Jul 29, 2025

Reporting Summary

Nature Portfolio wishes to improve the reproducibility of the work that we publish. This form provides structure for consistency and transparency in reporting. For further information on Nature Portfolio policies, see our Editorial Policies and the Editorial Policy Checklist.

Statistics

For all statistical analyses, confirm that the following items are present in the figure legend, table legend, main text, or Methods section.

n/a	Confirmed	
☐	☒	The exact sample size (*n*) for each experimental group/condition, given as a discrete number and unit of measurement
☒	☐	A statement on whether measurements were taken from distinct samples or whether the same sample was measured repeatedly
☐	☒	The statistical test(s) used AND whether they are one- or two-sided<br>*Only common tests should be described solely by name; describe more complex techniques in the Methods section.*
☒	☐	A description of all covariates tested
☒	☐	A description of any assumptions or corrections, such as tests of normality and adjustment for multiple comparisons
☐	☒	A full description of the statistical parameters including central tendency (e.g. means) or other basic estimates (e.g. regression coefficient) AND variation (e.g. standard deviation) or associated estimates of uncertainty (e.g. confidence intervals)
☐	☒	For null hypothesis testing, the test statistic (e.g. *F*, *t*, *r*) with confidence intervals, effect sizes, degrees of freedom and *P* value noted<br>*Give P values as exact values whenever suitable.*
☒	☐	For Bayesian analysis, information on the choice of priors and Markov chain Monte Carlo settings
☒	☐	For hierarchical and complex designs, identification of the appropriate level for tests and full reporting of outcomes
☒	☐	Estimates of effect sizes (e.g. Cohen's *d*, Pearson's *r*), indicating how they were calculated

Our web collection on statistics for biologists contains articles on many of the points above.

Software and code

Policy information about availability of computer code

Data collection	Zeiss AxioObserver Z1 epifluorescence microscopy system<br>ZEN Blue 3.11 (ZEN lite)<br>Huygens Professional deconvolution software<br>Image J/Fiji 1.54p<br>GDSC (Genome Damage and Stability Centre) Fiji plugins<br>Expert Line Easy3D STED microscope system (Abberior Instruments GmbH)<br>Olympus IX83 microscope<br>Imspector Software (Abberior Instruments Development Team, Imspector Image Acquisition & Analysis Software)<br>SequestHT search engine<br>Proteome Discoverer version 3.0.0.757 (Thermo Scientific)<br>AlphaFold version 2.3.230<br>PYMOL Version 2.2.2<br>AlphaPickle Version 1.5.4<br>GraphPad Prism software version 10.4.1
Data analysis	CENPA-PICH/CENPA-NUF2 coordinates: The measurements of the distance between foci in two channels of an image were done by using Spot Pair Distance Tool in FIJI software. The tool searches within a focus/box radius, typically± 5px, for a local maximum in the two pre-selected analysis channels. The centre-of-mass around each maximum, typically± 2px, is computed as the centre of intensity for each channel. Dragging from the clicked point creates a reference direction. The Euclidean distance between the centres is reported, optionally with the signed XY distance and angle relative to the reference direction. Visual guides are overlaid on the image to assist in spot selection and direction orientation. Available in the latest GDSC (Genome Damage and Stability Centre) Fiji plugins.

UFB intensity measurements: UFBs were stained for the UFB-binding complex components, including BLM, TOP3A and PICH following immunofluorescence protocol. FIJI software was used to determine the intensity of individual UFBs by drawing a scanline along the entire length of each UFB thread using PICH channel as a reference, which allowed the measurement of the absolute intensity of other channels corresponding to either BLM or TOP3A. The intensity of different channels was subjected to background correction prior to further analysis.

Centromere localisation analysis: The intensity of each centromere/cluster was determined using the Find Foci GUI Tool in FIJI software, which locates all the points of maximum intensity in the centromeric regions. This was used to generate a mask image containing all the pixels in the peak region above background in the CENPB-mCherry or PICH channel by using the Otsu thresholding method. The intensities of different channels within the centromeric masked regions were then measured using the Mask Analyser Channel Tool available in the latest GDSC (Genome Damage and Stability Centre) Fiji plugins.

For manuscripts utilizing custom algorithms or software that are central to the research but not yet described in published literature, software must be made available to editors and reviewers. We strongly encourage code deposition in a community repository (e.g. GitHub). See the Nature Portfolio guidelines for submitting code & software for further information.

Data

Policy information about availability of data

All manuscripts must include a data availability statement. This statement should provide the following information, where applicable:
- Accession codes, unique identifiers, or web links for publicly available datasets
- A description of any restrictions on data availability
- For clinical datasets or third party data, please ensure that the statement adheres to our policy

The datasets generated and/or analysed in the current study are available in the Source Data and in Figshare (https://doi.org/10.6084/m9.figshare.25818235). The mass spectrometry proteomics data have been deposited to the ProteomeXchange Consortium via the PRIDE69 partner repository with the dataset identifiers PXD064192 [http://proteomecentral.proteomexchange.org/cgi/GetDataset?ID=PXD064192], PXD052850 [http://proteomecentral.proteomexchange.org/cgi/GetDataset?ID=PXD052850], and PXD052669 [http://proteomecentral.proteomexchange.org/cgi/GetDataset?ID=PXD052669]. The authors declare that all data supporting the findings of this study are available within the paper and in the supplementary information files. Source data are provided with this paper. Microscopy image data acquired in the current study are often associated with specific experimental setups and the raw images and relevant details can be provided together upon reasonable requests.

Research involving human participants, their data, or biological material

Policy information about studies with human participants or human data. See also policy information about sex, gender (identity/presentation), and sexual orientation and race, ethnicity and racism.

Reporting on sex and gender	N.A.
Reporting on race, ethnicity, or other socially relevant groupings	N.A.
Population characteristics	N.A.
Recruitment	N.A.
Ethics oversight	N.A.

Note that full information on the approval of the study protocol must also be provided in the manuscript.

Field-specific reporting

Please select the one below that is the best fit for your research. If you are not sure, read the appropriate sections before making your selection.

☒ Life sciences ☐ Behavioural & social sciences ☐ Ecological, evolutionary & environmental sciences

For a reference copy of the document with all sections, see nature.com/documents/nr-reporting-summary-flat.pdf

Life sciences study design

All studies must disclose on these points even when the disclosure is negative.

Sample size	Sample size was comparative to previous cell biology-based studies of individual cell analyses, whilst also giving confidence of a fair statistical outcome. Our sample sizes ranged from at least 30 to several hundred in each experiment together with repetitions of two to five times. This range is typically used in a similar type of studies and can provide significant statistical tests. Smaller sample sizes (e.g. <30) were used in a few supplementary experiments because the experimental effects are huge, exhibiting over 4-fold differences or consistently detected in multiple different mutants.

Data exclusions	No data was excluded.
Replication	Experiments were repeated from two to five times and, if not, the measurements were repeated by using different cell lines and/or by measuring different subunits of the protein complexes. These generated consistent findings. In some cases, we also measured endogenous and ectopically expressed GFP-tagged proteins in different cell lines to ensure reproducibility of the data. In some experiments, we also compared staining between cell populations at different cell cycle stages using interphase cells as internal controls. All results were reproducible under the above measurements.
Randomization	Experiments were not randomized
Blinding	No specific blinding was taken during the experiments and analyses; however, repeats were usually completed independently without influence from other authors.

Reporting for specific materials, systems and methods

We require information from authors about some types of materials, experimental systems and methods used in many studies. Here, indicate whether each material, system or method listed is relevant to your study. If you are not sure if a list item applies to your research, read the appropriate section before selecting a response.

Materials & experimental systems

n/a	Involved in the study
☐	☒ Antibodies
☐	☒ Eukaryotic cell lines
☒	☐ Palaeontology and archaeology
☒	☐ Animals and other organisms
☒	☐ Clinical data
☒	☐ Dual use research of concern
☒	☐ Plants

Methods

n/a	Involved in the study
☒	☐ ChIP-seq
☐	☒ Flow cytometry
☒	☐ MRI-based neuroimaging

Antibodies

Antibodies used

Rabbit Aurora B, Abcam Cat#ab45145; RRID:AB_725589; IF:1:00
Mouse β-actin, Sigma, Cat# A5441; RRID: AB_476744; WB:1:5000
Rabbit BUBR1, Abcam, Cat# ab209998; IF: 1:100
Goat BLM (C-18), Santa Cruz, Cat# sc-7790; RRID: AB_2243489; IF:1:100
Mouse BLM (B-4), Santa Cruz, Cat# sc-365753; RRID: AB_10851630; IF:1:100
Rabbit BLM, Abcam, Cat# ab2179; RRID: AB_2290411; IF:1:100; WB:1:500
Goat BLM, Bethyl Lab, Cat#A300-120; RRID:AB_2779021; WB:1:2000
Mouse CENP-A, Abcam, Cat# ab13939; RRID: AB_300766: IF:1:100
Human Centromeres/CREST, Immuno Vision, Cat# HCT-0100; RRID: AB_2744669; IF:1:400
Mouse Cyclin B1, Santa Cruz, Cat#610219; RRID: AB_397617; IF:1:100
Alpaca Nanobody GFP-Atto488, Chromotek, Cat# gba488; RRID: AB_2631386; IF:1:200
Nanobody GFP-AlexaFluor488, Chromotek, Cat# gb2AF488-10; RRID: AB_2827573; IF:1:200
Rabbit GFP, Abcam, Cat# ab290; RRID: AB_303395; WB:1:2000
Rat GFP (3H9), Chromotek, Cat# 3h9-100; RRID: AB_10773374; WB:1:2000
Rabbit Ku80, Abcam, Cat# ab80592; RRID: AB_1603758;; WB:1:5000
Rabbit NUF2, Abcam, Cat# ab122962; RRID: AB_10902068; IF:1:200
Mouse PICH, Abnova, Cat# H00054821-B01P; RRID: AB_1573438; IF:1:100; WB:1:500
Rabbit PICH, Abnova, Cat# H00054821-D01P; RRID: AB_2262198; IF:1:100
Rabbit RIF1, Bethyl Lab, Cat# A300-568A; RRID: AB_669806; WB:1:1000
Rabbit RIF1, Bethyl Lab, Cat# A300-569A; RRID: AB_669804;WB:1:1000
Rabbit RPA70, Abcam, Cat# ab79398; RRID: AB_1603759; IF:1:200
Rabbit TOP3A, Abcam, Cat# ab108493;IF:1:100
Mouse SGO1, Abcam, Cat# ab58023; RRID: AB_945427;WB:1:500
Rabbit TOP3A, Proteintech, Cat# 14525-1-AP; RRID: AB_2205881; WB:1:500
Donkey anti-mouse AF488, Invitrogen, Cat# A-21202; RRID: AB_141607;IF:1:500
Donkey anti-mouse AF555, Invitrogen, Cat# A-31570; RRID: AB_2536180;IF:1:500
Donkey anti-mouse AF647, Invitrogen, Cat# A-31571; RRID: AB_162542;IF:1:500
Donkey anti-rabbit AF488, Invitrogen, Cat# A-31570; RRID: AB_2536180; IF:1:500
Donkey anti-rabbit AF555, Invitrogen, Cat# A-31572; RRID: AB_162543;IF:1:500
Donkey anti-rabbit AF647, Invitrogen, Cat# A-31573; RRID: AB_2536183;IF:1:500
Donkey anti-goat AF488, Invitrogen, Cat# 1463163; IF:1:500
Goat anti-human AF650, Abcam, Cat# ab98622; RRID: AB_10673586;IF:1:500
Goat anti-mouse STAR ORANGE, Abberior, Cat# ST580-1001; IF:1:200
Goat anti-rabbit STAR ORANGE, Abberior, Cat# ST580-1002; IF:1:200
Goat anti-mouse STAR RED, Abberior, Cat# STRED-1001; RRID: AB_2810982; IF:1:200
Goat anti-rabbit STAR RED, Abberior, Cat# STRED-1002; RRID: AB_2833015;IF:1:200
Goat anti-mouse HRP, Abcam, Cat# ab6789; RRID: AB_955439;WB:1:25000

	Rabbit anti-goat HRP, Agilent, Cat# P0160, RRID: AB_2636929;WB:1:60000<br>Goat anti-rat, HRP, ECL/Sigma Cat# GENA935;WB:1:25000<br>Donkey anti-Rabbit, HRP, ECL/Sigma Cat# NA9340, RRID: AB_772191;WB:1:40000
Validation	Validation data is provided in the corresponding manufacturer website. Validation is usually done by Western blotting using whole cell extracts or recombinant purified proteins, detecting the expected sizes of the target proteins. Immunofluorescent staining is also used to verify known localisation of the target proteins. The specificity of the antibodies are also tested in cell lines with depletion of the target proteins by RNAi or CRISPR KO, if available.

Eukaryotic cell lines

Policy information about cell lines and Sex and Gender in Research

Cell line source(s)	RPE1 hTERT (immortalized retinal pigment epithelial cells) ATCC Cat# CRL-4000, RRID: CVCL_4388<br>U2OS (Epithelial osteosarcoma) ATCC Cat# HTB-96, RRID: CVCL_0042<br>Hela (Epithelial-like adenocarcinoma) ATCC Cat# CCl-2; RRID: CVCL_0030<br>HCT116 (Colorectal carcinoma) ATCC RRID: CVCL_0291<br>HEK293T (Human epithelial embryonic kidney) ATCC RRID: CVCL_0063<br>HAP1 (Fibroblast-like chronic myelogenous leukaemia) ATCC RRID: CVCL_Y019<br>HAP1 BLM knockout (delta BLM) Prof. Marcel van Vugt (University of Groningen) (PMID: 26256213)<br>HAP1 deltaBLM + EGFP-BLM WT PMID: 31253795<br>HAP1 deltaBLM + EGFP-BLM Q672R PMID: 31253795<br>HAP1 deltaBLM + EGFP-BLM deleted first 51 residues Current study<br>HAP1 deltaBLM + EGFP-BLM deleted first 13 resides Current study<br>HAP1 deltaBLM + EGFP-BLM (N7A,N8A) Current study<br>HAP1 deltaBLM + EGFP-BLM (S144A) Current study<br>HAP1 deltaBLM + EGFP-BLM (S144E) Current study<br>HAP1 deltaBLM + EGFP-BLM (12A3E) Current study<br>HAP1 deltaBLM + EGFP-BLM (12A) Current study<br>HAP1 deltaBLM + EGFP-BLM (6A-1) Current study<br>HAP1 deltaBLM + EGFP-BLM (6A-2) Current study<br>HeLa EGFP-TOP3A + CENPB-mCherry-BLM Current study<br>HeLa EGFP-TOP3A + CENPB-mCherry-BLM (7A) Current study<br>HeLa EGFP-TOP3A + CENPB-mCherry-BLM (6A) Current study<br>HeLa EGFP-TOP3A + CENPB-mCherry-BLM (S17A,T20A) Current study<br>HeLa EGFP-TOP3A + CENPB-mCherry-RMI1 Current study<br>HeLa CENPB-mCherry-RMI1 Current study<br>HCT116 EGFP-TOP3A Current study<br>RPE1 hTERT EGFP-BLM Current study<br>RPE1 PICH-mAID-mClover3 (endogenously tagged) Current study<br>RPE1 hTERT deltaRIF1 Professor Steve Jackson (University of Cambridge) (PMID: 27079678)<br>RPE1 deltaRIF1 + EGFP-RIF1 WT Current study<br>RPE1 deltaRIF1 + EGFP-RIF1 PP1bs Current study
Authentication	All parental cell lines were authenticated by STR genotyping from European Collection of Cell Cultures.
Mycoplasma contamination	All cell lines were regularly tested by using Lonza Mycoplasma testing kit, and were negative for mycoplasma contamination.
Commonly misidentified lines<br>(See ICLAC register)	No ICLAC registered misidentified cell lines were used.

Plants

Seed stocks	N/A
Novel plant genotypes	N/A
Authentication	N/A

Flow Cytometry

Plots

Confirm that:

- [ ] The axis labels state the marker and fluorochrome used (e.g. CD4-FITC).
- [x] The axis scales are clearly visible. Include numbers along axes only for bottom left plot of group (a 'group' is an analysis of identical markers).
- [ ] All plots are contour plots with outliers or pseudocolor plots.
- [ ] A numerical value for number of cells or percentage (with statistics) is provided.

Methodology

Sample preparation	Cultured cells were trypsinised to prepare single-cell suspension before being fixed by 70% ice-cold ethanol. For cell cycle analysis, cells were re-suspended in standard propidium iodide (PI)/RNaseA FACS staining buffer.
Instrument	BD Accuri C6
Software	BD C Sampler
Cell population abundance	Over 95% cell purity was usually obtained and that was measured by FSC and SCC channels.
Gating strategy	Not gating was applied as the samples were generally very pure and lack of cell doublets.

- [ ] Tick this box to confirm that a figure exemplifying the gating strategy is provided in the Supplementary Information.
